# Trauma Exposure, Social Support and Mental Health in the General Population in Belgium

**DOI:** 10.3390/ejihpe14070136

**Published:** 2024-07-15

**Authors:** Emilie Muysewinkel, Lara Vesentini, Helena Van Deynse, Lydia Gisle, Pierre Smith, Helena Bruggeman, Johan Bilsen, Roel Van Overmeire

**Affiliations:** 1 Mental Health and Wellbeing Research Group, Vrije Universiteit Brussel, 1050 Brussels, Belgium; emiliemuysewinkel@hotmail.com (E.M.); lara.vesentini@vub.be (L.V.); johan.bilsen@vub.be (J.B.); 2 Department of Public Health, Vrije Universiteit Brussel, 1050 Brussels, Belgium; helena.van.deynse@vub.be; 3 Department of Epidemiology and Public Health, Sciensano, 1050 Brussels, Belgium; lydia.gisle@sciensano.be (L.G.); pierre.smith@sciensano.be (P.S.); helena.bruggeman@sciensano.be (H.B.)

**Keywords:** trauma, general population, depression, anxiety, social support

## Abstract

Introduction: International research has shown that trauma exposure can lead to mental health disorders and affect social support. However, there is little insight into trauma exposure and its related issues in the general population of Belgium. Methods: Secondary cross-sectional data on the general adult population were retrieved from the Belgian Health Interview Survey. Using a representative sample, data were collected on trauma exposure in the past 12 months, and the disclosure of trauma, social support, depressive/anxiety symptoms and background factors were investigated. Results: In total, 7728 participants were included in this study, and 4.8% (N = 369) reported trauma exposure within the past 12 months. People with trauma exposure (4.8%, N = 369) consistently had more anxiety and depressive symptoms than those without trauma (*p* < 0.001), and people with multiple trauma exposures had more symptoms than those with a single trauma (*p* < 0.001). Social support was observed to be lower among those with trauma exposure (*p* < 0.001), and 17.1% had never disclosed their trauma to anyone. Sexual violence was higher among women (64.5%) and was also the least disclosed form of trauma. Conclusions: Trauma exposure is associated with poorer mental health in Belgium. Around a fifth of those who experience trauma do not disclose their trauma, which is the most common after sexual violence.

## 1. Introduction

The Diagnostic and Statistical Manual of Mental Disorders (DSM)-5 refers to trauma as exposure to an event involving, for example, a threat to life, serious injury or sexual violence [1], while the International Codification of Diseases (ICD)-11 considers it as exposure to threatening or horrific events [2]. Thus, traumatic events include events such as disasters, physical violence, abuse, sexual violence, (armed) robberies, etc. [3,4]. Indirect exposure to trauma is, for example, media exposure to a violent event, which we do not take into account in this current study [1].

Around 70% of the world’s population are exposed to a traumatic event at least once in their life [5]. Trauma exposure is, therefore, quite common worldwide [6]. In some cases, experiencing trauma can lead to mental disorders such as depression, anxiety or post-traumatic stress disorder (PTSD) [5]. However, severe mental health issues after trauma are relatively less common, with most people not developing any mental health disorders and recovering quite quickly from any trauma they might experience [7]. For example, sexual violence is generally considered one of the traumata that have the highest associations with long-term PTSD. However, most studies show that while in the weeks following sexual violence, PTSD-like symptoms are present among 90% of those who endured sexual violence, 30–50% of those who experience it develop PTSD after three months [8], with 47% developing either moderate or severe depression after six months [9], which indicates that half do not develop PTSD or depression. Similarly, after the terrorist attacks of 9/11, for those living in Manhattan near the Twin Towers, depression rose by 9.7% one to two months later [10].

Furthermore, as in other types of traumata, it should also not be ignored that among those that do develop mental disorders, these issues are often long-term. For sexual assault, PTSD lasts, on average, nine years [8]; among the rescue workers who had worked during 9/11, 12.4% had PTSD two to three years after the attacks [10].

There are several factors that have been shown to decrease the development of mental health issues after trauma exposure, and one of the most important factors is social support [11,12,13].

People who receive help from others in times of need or perceive that others care for them will generally have fewer mental health issues after being exposed to trauma than people who do not have such social support [11,12,13]. The negative relation between perceived social support and trauma exposure is traditionally explained in three ways. First, those who are exposed to trauma and develop mental health issues exclude their social support [14]. Second, social support excludes the victim of trauma for whatever reason [15,16]. One possibility for this is, for example, that the social environment has no time to provide a listening ear to a person with trauma or because the trauma exposure is associated with stigma and victim blaming [15]. Third, it is also possible, of course, that those experiencing trauma have no social support as a buffer to begin with [12,13]. Qualitative studies have shown that people exposed to violence or sexual assault can experience both a form of social self-exclusion and a situation where sources of social support do not wish to support someone if their view of them has changed too much (e.g., moodier or aggressive behavior or more anxiety in the victim) [16,17] Negative reactions from one’s social environment can lead to worse mental health outcomes, and general negative societal acknowledgment will lead to less intent to disclose [15,18,19]. 

Nevertheless, the possibility of disclosing one’s experience of trauma is important in order to help avoid the development of long-term mental health issues [18]. Disclosing means that someone who was exposed to trauma can talk about the trauma to someone else, whether this support is informal (e.g., friends, family or partner) or formal (e.g., the police or a mental health worker).

However, the benefits of both social support and disclosing are not always evident. Victims of trauma might push away social support, or people previously offering support can terminate the relationship with a victim [18]. Similarly, disclosing might not happen if the victim is ashamed, as is often the case for victims of sexual violence [17,18,20,21].

Thus, insight into trauma exposure is an important public health priority. Yet, to our knowledge, there are hardly any representative estimates on trauma exposure in the general population of Belgium and on the characteristics of those exposed. There has been an estimation of 2.6% of the population having lifetime post-traumatic stress disorder [22], but there are no indications of overall trauma exposure.

Yet, there are important reasons to investigate the manner of exposure to trauma in Belgian society. There is generally little attention paid to PTSD in Belgium and, consequently, to trauma exposure. One example of this is that only since 2017 have there been sexual assault care centers in Belgium. This is also reflected in the poor state of Belgian psychotraumatology, with even government reports noting the lack of development in this field in Belgium [23,24]. 

In this current study, we will look at the prevalence of trauma, the extent to which it is disclosed, perceived social support and the association with mental health issues in a representative sample of the general population in Belgium in 2018. We will concentrate on direct exposure to trauma rather than indirect, as people who have experienced this type of exposure have the highest risk of developing mental health issues, and it is thus the most relevant to study [5].

## 2. Materials and Methods

### 2.1. Data Collection and Population

Data were extracted from the 2018 Health Interview Survey (HIS) carried out by Sciensano, the Belgian Institute for Health. Using a representative sample, the Belgian HIS collects self-reported information on the health of the general population. Participants were selected from the national register using a multistage stratified cluster sample. Details on the methodology have been published elsewhere [25]. Face-to-face interviews were conducted. The National Registry of Belgium was used to construct a sampling framework, with the aim of establishing a representative sample of the population. In total, 10,700 respondents were interviewed.

In this current study, we selected all respondents aged 15 and up, as those younger than this did not receive questions on trauma exposure. Furthermore, we excluded respondents that had not answered questions on trauma exposure. In the Section 3, we have differentiated between those with trauma exposure and those without.

### 2.2. Measures

Sociodemographic factors included gender and age (continuous).

Participants were asked if they had been victim of any kind of violence in the past 12 months, and if so, they indicated, from a list of events, which one(s) they had experienced. In this study, trauma exposure is operationalized as having been a victim of robbery, armed robbery, burglary, physical violence or sexual violence in the past 12 months. From the list of violent events, only “verbal violence” was excluded, as this did not meet our definition of trauma exposure.

A binary variable called “overall trauma” was created and took into account exposure to any of those traumatic events. Throughout the text, we will refer to having experienced a traumatic event as “trauma exposure”. The group was divided into “single trauma” for people who only had experienced one type of trauma and “multiple trauma” for those who experienced more. The location where the reported event took place was also probed—at home, at work/school, at a public place/road, or elsewhere.

Social support was measured using the Oslo-3 scale [26]. These are three questions on social contacts and include questions such as “How many people are so close to you that you can count on them if you have serious personal problems?” The scale ranges from 0 to 11 and can be divided into three categories: 0–5: poor social support; 6–8: moderate social support; and 9–11: strong social support. Social support was only used categorically and descriptively but was used continuously when using tests (α = 0.714).

People who had experienced trauma were asked whether they had disclosed the trauma, for which the terms “contacting or consulting” were used in the questionnaire. The full question was “As a result of this act of violence, did you consult or contact with one or more of the following persons, services or institutions?” A distinction was made between informal disclosure (family, friends, trustees at work/school), formal disclosure (police, medical services, psychologists, law or juridical agencies, victim support, call-centers for assistance) or not disclosing to anyone.

Finally, to investigate mental health in relation to trauma, two common mental health measures were used. First, depressive disorders were measured using the Patient Health Questionnaire—9 items (PHQ-9) [27]. Second, anxiety disorder was measured using the Generalized Anxiety Disorder—7 items (GAD-7) [28]. The PHQ-9, which ranges from 0 to 27, can also be categorized, where 0–4 is no depression, 5–9 is mild depression, 10–14 is moderate depression, 15–19 is moderately severe depression and 20 and up is severe depression (α = 0.869). The GAD-7 ranges from 0 to 21, where 0–4 is no anxiety, 5–9 is mild anxiety, 10–14 is moderate anxiety and 15 to 21 is severe anxiety (α = 0.898).

### 2.3. Analysis

Descriptive statistics were mainly used to describe the trauma-exposed group. To estimate the relation between trauma, social support and mental health in the general population, a linear regression analysis was used, with the severity of depressive and anxiety symptoms as outcomes, while controlling for gender, age, trauma exposure and social support. For the group of trauma-exposed people, a linear regression analysis was used, with depression and anxiety as dependent variables (both as continuous variables), and disclosure (binary), social support (continuous), gender and age as independent variables.

To avoid multiple testing problems, Bonferroni corrections were applied, and *p*-values were significant if *p* ≤ 0.0125.

### 2.4. Ethics

This study was approved by the ethics committee of the Ghent university hospital (Commissie voor Medische Ethiek), B.U.N.: B6702020000031. All participants were informed that their information would be used in scientific research.

## 3. Results

### 3.1. Total Sample Characteristics

We included 7728 people, of which 52.4% were women, and the average age was 50.4 (±18.5) with a range of 15 years to 101 years. The social support score was, on average, 7.5 (±2.1), with 16.5% of the people having poor social support, 48.6% having moderate social support and 33.7% having strong social support.

In total, 4.8% (N = 369) experienced some form of traumatic event in the past 12 months, with the most common being burglary/robbery/armed robbery (71.3% among those having experienced trauma; 3.4% in the total population) (see Table 1). The most common place to experience trauma exposure was at home (39% of those who had trauma exposure; 1.8% in the total population). Of these 4.8% who had trauma exposure, 175 (47.4%) had experienced multiple traumata.

### 3.2. Trauma Exposure According to Age and Gender

On average, people who had experienced overall trauma were younger (M = 45.9; ±18.7 vs. M = 50.7; ±18.4). More women had trauma exposure than men (54.7% vs. 45.3%). Among those with trauma exposure, more women had multiple trauma exposure than men (53.7% vs. 46.3%).

### 3.3. Disclosure

In total, 17.1% of participants exposed to trauma never disclosed it to anyone. Among participants who disclosed it, the most common informal contact(s) to disclose to were family members (29.5%) and friends (26.0%). Formal disclosure most commonly occurred to the police (47.2%).

The age group that disclosed the least were those between 18 and 25 years old, with 20.8% not having disclosed the trauma. Regarding gender, males disclosed less than females (26.7% vs. 22.3%).

Non-disclosure after trauma was the most common after sexual violence (see Table 2). Almost twice as many women than men had experienced sexual trauma (64.5% and 35.5%, respectively). In both cases, it was often not disclosed (27.4% for sexual violence), and women disclosed less often than men; 70.6% females did not disclose vs. 29.4% males. Those who had experienced multiple traumata disclosed less often than those who had experienced a single event of trauma (21.1% vs. 13.4%).

### 3.4. Social Support

Those with trauma exposure ($\bar{X}$ = 7.0 ± 2.2) had lower levels of social support than those without trauma exposure ($\bar{X}$ = 7.5 ± 2.1). People with multiple trauma exposure had a lower level of perceived social support ($\bar{X}$ = 6.7 ± 2.1) than those with single trauma exposure ($\bar{X}$ = 7.3 ± 2.2).

### 3.5. Mental Health

Overall, people with trauma exposure had more severe depressive symptoms ($\bar{X}$ = 5.3 ± 5.3 vs. $\bar{X}$ = 3.4 ± 4.3) and more severe anxiety symptoms ($\bar{X}$ = 5.3 ± 5.0 vs. $\bar{X}$ = 3.7 ± 4.2) than the non-exposed. Of those who had trauma exposure, 58.1% did not screen positive for depression, 21.9% had mild depression, 11.8% had moderately severe depression and 5.9% had severe depression. For those without trauma exposure, this was significantly lower; 73.6% did not screen positive for depression, 17.4% had mild depression, 5.9% had moderately severe depression and 1.9% had severe depression.

Roughly the same was found regarding anxiety. For those with trauma exposure, 54.2% had no anxiety, 26% had mild anxiety, 14% had moderate anxiety and 5.9% had severe anxiety. For those without exposure, 67.7% had no anxiety, 21.9% had mild anxiety, 7.1% had moderate anxiety and 3.3% had severe anxiety (see Table 3).

For specific trauma exposure (e.g., sexual violence, physical violence), both depression and anxiety were consistently and significantly higher in those with trauma exposure than those without trauma exposure. This was highest for physical violence and violence at home (see Table 4).

### 3.6. Traumatic Events, Social Support and Mental Health

A linear regression analysis showed that in a model with depressive symptoms as the outcome, as well as gender, age, having experienced a traumatic event and social support, all variables were significant, as was the model (*p* < 0.001; R = 0.371; adjusted R^2^ = 0.137). Having experienced trauma, having lower levels of social support, being female and being younger were significantly associated with experiencing more depressive symptoms. Similarly, for anxiety as the outcome, all variables were, again, significant, with the same interpretation again (*p* < 0.001; R = 0.345; adjusted R^2^ = 0.119) (see Table 5).

Among those with trauma exposure, a linear regression analysis was performed with gender, age, disclosure, single/multiple trauma and social support as predictors. In this model, higher levels of social support were shown to decrease depressive symptoms, while having multiple traumata increased it (*p* < 0.001; R = 0.430; R^2^ = 0.173). The same interpretation applied to the model with anxiety the as outcome. (*p* < 0.001; R = 0.398; R^2^ = 0.146).

## 4. Discussion

In this current study, we investigated a representative sample of the general population in Belgium for trauma exposure, disclosure, mental health and social support. The most common trauma experienced in the 12 months prior to the survey was burglary or (armed) robbery. Trauma exposure is associated with a higher severity of symptoms and a prevalence of depression and anxiety, as well as lower social support. Those who had experienced more than one trauma in the last twelve months had more depressive and anxiety symptoms than those with single trauma exposure, and they had lower levels of social support. Of those having experienced a traumatic event, 17.1% did not disclose the event to anyone. Not disclosing was the most frequent after sexual violence. For those who did disclose, the police or family were the most common disclosees. However, disclosing was not associated with fewer depressive/anxiety symptoms.

As in other studies, more than half of the people exposed to trauma had no depressive or anxiety symptoms [8,9,10]. Naturally, sexual violence, physical violence and other traumata should not be minimized in their non-mental disorder impact (e.g., social impact and stigma). Furthermore, it is quite clear that people who have experienced trauma not only have a higher chance of developing mental disorders but additionally have a higher chance of suffering from social isolation [13]. This was also seen in this current study, where perceived social support was lower among those with trauma exposure and even lower among those with multiple traumata.

A general negative societal acknowledgement of trauma and negative reactions from a social environment will lead to worse mental health outcomes, as well as less intent to disclose [15,18,19]. This might be why sexual violence is less often disclosed in this current study, as it can be related to more (self-) stigma and taboo. Regardless of the direction the loss of social support takes, it was clear in this current study that those with trauma exposure indeed perceived less social support.

While disclosing was found to be important in other studies to decrease mental health issues [15,18], the issue seems to be that experiencing more trauma exposure will also lead to being less likely to disclose. This can indicate a form of habituation and/or a loss of trust in institutions (which is already quite low in Belgium) and a loss of trust in informal support [29]. A further concern is that 1/5 of those exposed to trauma did not disclose the trauma to anyone, and almost half of these were 25 years old and younger. For sexual violence, studies have shown that a lack of disclosure can often be related to a variety of reasons, such as cultural taboo or shame or a fear of the reaction of people [17]. However, having the perception of having strong social support was found to be more important as a buffer against depressive symptoms and anxiety symptoms than disclosure. Thus, for those exposed to trauma, knowing that people support them is a better buffer than simply reporting or talking about the trauma exposure.

### 4.1. Limitations and Strenghts of This Study

There are several limitations in this study. The first limitation is that our group of people who had experienced trauma was relatively small. While this is certainly positive from a societal viewpoint, this did not allow us to look at a specific traumatic event such as sexual violence in relation to higher or lower levels of social support. The second limitation is that trauma can be seen as broader in definition than how it was operationalized in this study. We took its form of “direct” exposure, while the DSM-5 would also include, for example, seeing the remains of people after a violent event [1]. Additionally, it is not always clear whether exposure to robbery would always fit the criteria for trauma [1,2]. Furthermore, we measured trauma only for a 12-month period and not life-time prevalence. The third limitation is that, as with all cross-sectional studies, our study cannot show causal relations. Finally, our study had a cut-off for age of 15 years old for both the entire sample and the subsample of people with trauma exposure. The reason for this is because data collected for ages younger than 15 required parents to be present and would be, thus, not self-reported anymore.

Our study is one of the first to explore the link between disclosure, social support, trauma exposure and its psychosocial consequences in a representative sample. Moreover, this is the first study to assess this topic for the general population in Belgium. While most studies have focused on PTSD, this study has given rates for depression and anxiety, which are understudied compared to PTSD in relation to trauma exposure. Finally, to our knowledge, there are also no studies that look this closely into trauma disclosure.

### 4.2. Recommendations

Our findings give rise to a couple of recommendations. First, from a societal viewpoint, the relatively low rate of disclosing after sexual violence is concerning. While countries in Europe have increased the number of sexual assault centers, this current study’s data were collected at a time when sexual assault centers in Belgium were relatively new. Either way, public health campaigns should continue to decrease the stigma and possible cultural taboo related to sexual violence. Second, public health policies should facilitate victim support groups. It is clear from other studies that such groups can help to create a sense of social support (e.g., [16]). Third, research should be performed on the barriers younger people experience when disclosing their trauma. Finally, as police are often contacted for disclosure (e.g., legal reasons), it is important to investigate the quality of psychosocial support police provide for victims.

## 5. Conclusions

Trauma exposure is associated with poorer mental health in Belgium. Around a fifth of those experiencing trauma had not disclosed their trauma, which was most common after sexual violence.

## Figures and Tables

**Table 1:** Characteristics of the total sample (N = 7728) and trauma sample (N = 369).

	N	% for Total Sample	N for Overall Trauma Exposure	% for Overall Trauma Exposure
Sex				
Men	3682	47.6	167	45.3
Women	4046	52.4	202	54.7
Overall trauma exposure				
Yes	369	4.8		
No	7359	95.2		
Single and multiple trauma				
Single			194	52.6
Multiple			175	47.4
Type of trauma (N = 369) *				
Burglary, robbery, armed robbery	263	3.4		71.3
Physical violence	162	2.1		43.9
Sexual violence	62	0.8		16.8
Place of trauma occurrence				
Violence at home	151	2.0		40.9
Violence at work/school	77	1.0		20.9
Violence at Public place/public road	165	2.1		44.7
Violence Elsewhere	92	1.2		24.9
If you experienced violence, did you disclose to… (N = 369) *				
Family	109	1.4		29.5
Friends	96	1.2		26.0
Trustee at work/school	24	0.3		6.5
Police	174	2.3		47.2
Medical service	29	0.4		7.9
Psychologist	38	0.5		10.3
Lawyer, law agency	17	0.2		4.6
Victim assistance	16	0.2		4.3
Call-center	6	0.1		1.6
Consulted someone else	18	0.2		4.9
Did not consult anyone	63	0.8		17.1

* Multiple answers were possible.

**Table 2:** Having experienced trauma and consulting someone.

	Did Not Disclose	%	Disclosed	%
Burglary, robbery, armed robbery	40	15.2	223	84.8
Physical violence	33	20.4	129	79.6
Sexual violence	17	27.4	45	72.6
Violence at home	16	10.6	135	89.4
Violence at work/school	19	24.7	58	75.3
Violence at public places	32	19.4	133	80.6
Violence elsewhere	20	21.7	72	78.3

**Table 3:** Categories of depression and anxiety severity.

	% Trauma Exposed (N = 369)	% Non-Trauma Exposed (N = 7359)
Depression categories		
No symptoms	58.1	73.6
Mild depression	21.9	17.4
Moderately severe depression	11.8	5.9
Severe depression	5.9	1.9
Anxiety categories		
No symptoms	54.2	67.7
Mild anxiety	26	21.9
Moderate anxiety	14	7.1
Severe anxiety	5.9	3.3

**Table 4:** Mental health according to the form of trauma exposure.

	Depression Symptoms		Anxiety Symptom	
	M	SD	M	SD
Overall trauma				
Yes	5.31	5.26	5.35	4.97
No	3.45	4.30	3.74	4.26
Burglary, robbery, armed robbery				
Yes	4.62	4.95	4.78	4.67
No	3.50	4.35	3.79	4.29
Physical violence				
Yes	6.69	5.90	6.45	5.28
No	3.48	4.31	4.27	4.27
Sexual violence				
Yes	5.90	5.38	6.44	5.37
No	3.52	4.36	3.80	4.29
Violence at home				
Yes	6.06	5.80	5.94	5.29
No	3.47	4.30	3.76	4.26
Violence at work/school				
Yes	5.43	5.07	5.67	4.57
No	3.71	4.46	4.08	4.38
Violence at public place				
Yes	5.79	5.27	5.97	5.08
No	3.45	4.31	3.73	4.25
Violence elsewhere				
Yes	6.15	5.73	6.09	4.91
No	3.49	4.33	3.78	4.29

People with multiple trauma exposure also had more severe depressive ($\bar{X}$ = 6.7 ± 5.8 vs. $\bar{X}$ = 4.1 ± 4.4) and anxiety symptoms ($\bar{X}$ = 6.6 ± 5.3 vs. $\bar{X}$ = 4.2 ± 4.3) than those with single trauma exposure.

**Table 5:** Regression analysis with the severity of depressive symptoms and severity of anxiety symptoms.

	B	SD	*p*	CI	Adjusted R^2^
Model 1: depressive symptoms in full sample			<0.001		
Gender (ref. male)	1.120	0.094	<0.001	0.935, 1.305	
Age	−0.023	0.003	<0.001	−0.029, −0.18	0.137
Trauma (ref. no)	1.372	0.222	<0.001	0.936, 1.808	
Social support	−0.688	0.022	<0.001	−0.732, −0.644	
Model 2: anxiety symptoms in full sample			<0.001		
Gender (ref. male)	1.312	0.094	<0.001	1.127, 1.497	
Age	−0.031	0.003	<0.001	−0.036, −0.026	0.119
Trauma (ref. no)	1.147	0.221	<0.001	0.714, 1.581	
Social support	−0.576	0.022	<0.001	−0.620, −0.532	
Model 3: depressive symptoms in trauma-exposed sample			<0.001		
Gender (ref. male)	0.653	0.513	0.217	−0.374, 1.645	
Age	−0.020	0.014	0.143	−0.048, 0.007	0.173
Single/multiple trauma (ref. single)	1.955	0.532	<0.001	0.908, 3.002	
Social support	−0.879	0.123	<0.001	−1.121, −0.637	
Disclosure (ref. yes)	0.378	0.678	0.578	−0.956, 1.712	
Model 4: anxiety symptoms in trauma-exposed sample			<0.001		
Gender (ref. male)	0.596	0.490	<0.001	−0.367, 1.560	
Age	−0.026	0.013	<0.001	−0.052, 0.000	0.146
Single/multiple trauma (ref. single)	1.782	0.509	<0.001	0.782, 2.783	
Social support	−0.734	0.117	<0.001	−0.965, −0.503	
Disclosure (ref. no)	0.260	0.649	<0.001	−1.017, 1.536	

Social support and age were used continuously.

## Data Availability

The data used were secondary data, which can be requested from Sciensano directly.
